# Leveraging strain competition to address antimicrobial resistance with microbiota therapies

**DOI:** 10.1080/19490976.2025.2488046

**Published:** 2025-04-07

**Authors:** Danielle Barrios Steed, Dylan Koundakjian, Anthony D. Harris, Adriana E Rosato, Konstantinos T Konstantinidis, Michael H Woodworth

**Affiliations:** a Department of Medicine, Division of Infectious Diseases, Emory University School of Medicine, Atlanta, GA, USA; b Department of Medicine, Emory University Hospital, Atlanta, GA, USA; c Department of Epidemiology & Public Health, University of Maryland School of Medicine, Baltimore, MD, USA; d Institute for Healthcare Computing, University of Maryland, Baltimore, MD, USA; e Center for Molecular Medicine, MaineHealth Institute for Research, Scarborough, ME, USA; f School of Civil and Environmental Engineering, Georgia Institute of Technology, Atlanta, GA, USA

**Keywords:** Antibiotic resistance, fecal microbiota transplantation, microbiota, microbiome, bacterial competition, quorum sensing, bacteriocins

## Abstract

The enteric microbiota is an established reservoir for multidrug-resistant organisms that present urgent clinical and public health threats. Observational data and small interventional studies suggest that microbiome interventions, such as fecal microbiota products and characterized live biotherapeutic bacterial strains, could be an effective antibiotic-sparing prevention approach to address these threats. However, bacterial colonization is a complex ecological phenomenon that remains understudied in the context of the human gut. Antibiotic resistance is one among many adaptative strategies that impact long-term colonization. Here we review and synthesize evidence of how bacterial competition and differential fitness in the context of the gut present opportunities to improve mechanistic understanding of colonization resistance, therapeutic development, patient care, and ultimately public health.

## Antimicrobial resistance is a global challenge due to diminishing numbers of effective therapies

Antimicrobial resistance (AMR) threatens medical care for sepsis, cancer, surgical indications, and transplantation that routinely require safe and effective antibiotics. Global burden analyses estimate nearly five million deaths were associated with bacterial AMR in 2019^1^ and treatment of AMR infections just in the United States were estimated to cost $4.6 billion in 2017.^2^ Motivated by these challenges, a joint FDA-CDC workshop in August 2022 emphasized the need to develop novel infection prevention strategies for AMR bacteria, particularly approaches that do not select for increased AMR. Several excellent reviews have highlighted the disruptive effects of antibiotic exposure on the gut microbiome,^3^ trends in AMR epidemiology, clinical management of gram negative AMR infections,^4^ and touchpoints where microbiome therapies could reduce AMR bacterial colonization.^5^ A consistent theme is that the gut microbiome holds potential for urgently needed infection prevention strategies.

Notably, genes associated with the most extensive AMR phenotypes are carried by a minority of strains within diverse species. Although the most clinically significant AMR genes are well-characterized, especially in prevalent and well-studied pathogens, many AMR genes are less well understood. Some genes that have been classified as AMR genes do not increase phenotypic antibiotic resistance of clinical isolates in vitro or have clear association with clinical treatment failures.^6,7^ Although AMR genes on mobile elements can transfer readily to related taxa, the potential for many AMR genes to expand to commensal bacterial species within the gut appears more limited but remains largely unknown.^8,9^ Identifying the factors needed to restrict the invasion, persistence, and dominance of AMR strains presents an unrealized opportunity to reduce the prevalence and ongoing transmission of priority AMR genes. Crucially, many of these competition and colonization ecological strategies also likely underpin engraftment dynamics of therapeutic strains and bacterial carrying capacity of an anatomic site.^10^

Thus, microecology dynamics both from the perspective of AMR genes and strains but also of human microbiota contexts have distinct value for developing novel interventions to reduce AMR for patients and populations. Strain-specific strategies may provide a mechanistic framework for the next generation of safe and effective microbiome therapies for AMR beyond fecal microbiota transplantation (FMT). To support this goal, we review work that clarifies how bacterial competition can impact microbiota composition and pathogen colonization. This review also highlights work that has advanced data-driven bacterial taxonomic definitions below the level of species and bioinformatic tools that are required to track dynamics of bacteria, even closely related isolates of the same species within complex human microbial populations.

## Intestinal colonization with AMR pathogens is associated with important health outcomes

The intestinal tract is recognized as a reservoir for pathogens and the potential accumulation of genetic AMR determinants. Notably, three of the five ‘Urgent Threats’, and eight of the eleven ‘Serious Threats’ classified by the 2019 CDC AMR Threats Report are MDROs that can establish intestinal colonization. Successes in the translational development and FDA approval of microbiota therapies could propel development of novel therapies to reduce intestinal colonization with these prioritized AMR bacteria.

Several observational studies have identified a significant association of increased pathogen intestinal relative abundance with increased risk of subsequent bloodstream and urinary tract infection.^11–13^ These data suggest that reducing pathogen relative abundance could have meaningful clinical benefit.

Notably, these studies inferred isolate representation by 16S rRNA gene amplicon taxonomic classification or sequence identity with isolate genomes and predated many shotgun metagenomic analytic methods that could more precisely map gut microbiome data to isolate genomes. Relative abundance thresholds that were predictive of infection from these studies thus may benefit from further analysis with shotgun metagenomic approaches and validation in broader populations beyond hematopoietic stem cell transplant recipients and long-term care facility residents.

## Trials of fecal microbiota transplantation to reduce AMR bacterial colonization demonstrate encouraging, yet variable efficacy

Some of the most supportive data for reduced AMR bacterial colonization after fecal microbiota transplantation (FMT) are from secondary analyses of FMT-treated patients with recurrent *C. difficile* infection (RCDI) or hematologic malignancies with extensive antibiotic exposures.^14,15^ Similarly, secondary analyses of microbiota therapeutics that are FDA approved for prevention of RCDI (fecal microbiota spores, live-brpk (Vowst) and fecal microbiota, live-jslm (Rebyota)) suggest that they also may reduce the burden of many clinically significant AMR genes.^16,17^ Arguably, a primary lesion in recurrent *C. difficile* infection is impaired microbial bile acid metabolism, which also results in dramatic shifts in microbiome composition. These large compositional shifts are easily detectable with relatively coarse 16S rRNA gene variable subunit amplicon sequencing analyses. However, studies that have focused more directly on efficacy of FMT to reduce colonization with MDROs other than *C. difficile*, particularly those that leveraged metagenomic approaches, have shown that many MDRO colonized patients may not necessarily have large compositional dissimilarity compared to healthy controls and revealed potential effects of FMT that may not have been anticipated.

Studies that focus on FMT for MDRO decolonization with the gold standard of isolate culture have reported variable efficacy, ranging from 38% to 88%.^18^ Interestingly, one study showed that FMT receipt can reduce the frequency of infection and healthcare utilization measured by days of antibiotic use and length of stay, even among patients who remain MDRO culture positive.^19,20^ We recently reported results of PREMIX, a randomized, controlled trial of renal transplant recipients who were colonized with AR bacteria and treated with FMT by retention enema. At last study visit, 8/10 FMT-treated participants had negative stool cultures and PREMIX participants had longer time to recurrent AMR bacterial infection compared to contemporaneous controls.^21^ In PREMIX, pathogen abundance was relatively low and many participants’ microbiome composition was similar to the stool donor at baseline indicating more subtle differences than those seen in recurrent *C. difficile*. Other studies of single-dose FMT and upper routes of FMT administration have reported more modest efficacy for MDRO decolonization.^22^ Meta-analyses of publicly available metagenomic data indicate that conditioning regimens before FMT treatment can have important effects on the donor strains that are detected at follow-up time points.^23,24^ A key example of the importance of dose was the SER-109 phase two trial that did not meet its endpoints but in post-hoc metagenomic analysis was determined to be related to dosing.^25^ The product met endpoints in a phase 3 trial with a higher dose that ultimately supported FDA approval.^26^

A clear, but even less studied, signal from FMT (and fecal microbiota, live-jslm) clinical trials is that several genes are transferred from donors to recipients that are classified as AMR genes.^16,27^ Rashidi et al^27^ studied the AMR gene dynamics of 100 allogeneic hematopoietic cell transplant patients treated with FMT. They found that within the first few weeks in the early post-FMT that AMR gene transfer is largely associated with administered donor taxa and that long-term resistance to new AMR gene acquisitions develops as stable communities form in the later period.^27^ To date, with the exception of the transfer of ESBL strains reported in one study, there are little data to suggest that donor-derived AMR genes transferred by FMT have meaningful risk to recipients^28^ but this question is largely unresolved. These observations highlight the value of FMT studies as a model to understand clinical effects of AMR genes in diverse clinical contexts.

Together, these data indicate the potential benefit of microbiome interventions for AMR bacterial colonization. However, the field is still at the beginning of understanding the impact of FMT and other microbiota therapies across states of microbiome disruption, disease states, preparations, and routes of administration. For example, some patients who have alpha diversity that is comparable to healthy controls may require conditioning with antibiotics or bowel preparation regimens to support engraftment of therapeutic strains. Other patients with recurrent *C. difficile* or extensive antibiotic exposures for other indications may not require pre-treatment with additional antibiotics or bowel preparation regimens for therapeutic strains to engraft and persist. To realize effective microbiome interventions for AMR, extensive work is needed to optimize dosing, routes of administration, recipient conditioning with antibiotic or bowel preparation regimens, and ecological dynamics that are required for optimal efficacy – perhaps even for specific colonizing MDRO taxa (e.g. *Enterobacterales, Pseudomonas spp, Enterococcus spp*). Several recent advances in classifying bacteria below the level of species with high throughput metagenomic methods have expanded the toolkit to complete this work.

## Genomovars and strains

Development of improved gut microbiome diagnostics and therapies for MDRO colonization requires recognition of diversity within bacterial taxonomic groups. For example, *Escherichia coli* includes strains that are used as probiotics (*E. coli* Nissle 1917) and chassis for delivery of diagnostic or therapeutic gene products,^29,30^ a highly prevalent healthy microbiota constituent without virulence factors,^31^ and a cause of foodborne outbreaks.^32,33^ Accordingly, gene content diversity can be substantial within species. Genomes of the same species may vary by up to 30–35% of the total genes in the genome, and these genes often include important virulence or competition factors.^34^ This species diversity is intuitive to clinicians that await antibiotic susceptibility testing to inform final treatment decisions for specific isolates but many other important strain-specific phenotypes also likely have underexplored clinical relevance for microbiota invasion, long-term colonization, shedding or environmental persistence, and virulence. Therefore, there is distinct value in obtaining genome- (not just species-) level resolution in clinical microbiome studies or interventions. This diversity below the classification of species underscores the limitation of microbiome-profiling with 16S rRNA gene subunit amplicon compositional analyses, which does not reliably classify below the genus level and provides no information about antibiotic susceptibility within populations.

Note that while plasmid DNA, which commonly carries ARGs, can be assembled based on short, or even more efficiently, long-read sequencing, it often remains challenging to unequivocally link such plasmids to a genome based solely on assembly, and/or genome binning. Indeed it has been shown in recent literature that plasmids are often mis-binned to a genome or not binned at all due to their different copy number (abundance) relative to the rest of the genome and/or high frequency of sequence repeats.^35,36^ Sequencing technologies that are able to link independent DNA molecules of a cell, such as chromosome conformation capture (Hi-C) or techniques like Epic-PCR could be used instead if linking plasmids to a genome is viewed as necessary.^37,38^ Shotgun short read, hybrid short read and long read, and Hi-C metagenomic sequencing methods are useful for detection and quantification of AMR genes but highlight challenges of how to consistently classify important differences within species-level populations.

Recent work has focused on this question of classification of genomes and isolates within species. Although this framework of genomic classification is nascent and under development, it has important utility for basic and translational research. Strains have been traditionally defined as descendants of the same cultured isolates but the application of this definition to natural (gut) populations has been less straightforward.^39^ Further, gut microbial populations of a certain species may be typically composed of hundreds of strains, each carrying a large number of strain-specific genes but a precise definition of strain remains elusive, especially for natural (metagenomic) populations. A recent survey of the ~ 300 best-sampled bacterial species by isolate genomes revealed a consistent gap in relatedness values, measured by genome-average nucleotide identity (ANI) or another metric, that can be used to define natural units (or clusters) of genomes within species. Specifically, the analysis showed a shortage of ANI values, by 3-fold or more, between 99.2% and 99.8% (average around 99.5%) compared to > 99.8% or < 99.2% ANI.^40^ The 99.5% ANI-based units appear to be most similar to the Sequence Types (STs)^41^ among existing intraspecies categories but provide clusters of genomes that are at least 20% more homogenous than the current ST-based practice.^40^ Therefore, the intra-species diversity appears to be organized in sequence-discrete units, similar to the species-level diversity noted previously.^42,43^ Rodriguez-R et al., proposed the name genomovar to refer to these natural intraspecies units. Examining the results of previous studies has showed that genomovars often correspond to ecologically and/or epidemiologically distinct groups of genomes such as those causing local enteric outbreaks.^44–46^ No other ANI gap was observed within species. Hence, it was proposed to define strains at a higher level of relatedness based on the expectation by taxonomists that members of the same strain should represent highly similar organisms in terms of phenotype as derivatives of the same culture.^39^ Specifically, it was proposed to define stains with > 99.99% ANI, which typically corresponds to > 99% shared gene content.^46^ It should be noted that the ANI thresholds mentioned above are not strict standards but rather general guidelines that appear to work in most but not necessarily all cases. In fact, they may require adjustment to match diversity patterns for species of interest.^40^ Notably, similar 99.5% ANI distinct units within prokaryotic and eukaryotic viral species^47^ and pathogenic protozoa^48^ have been observed more recently, indicating that the diversity patterns reported above for bacteria may be more broadly applicable to additional microbes.

## Metagenomic methods to track genomovars in complex populations

The identification of sequence-discrete units within species that are ecologically and/or functionally distinct opens new possibilities to study the microbiome at a level that matters for phenotype, that of the individual, discrete population (or genomovar) within a species. To identify these units, it will be necessary to obtain the ANI value distribution among enough genomes of the species in question (typically over 20 to 30 genomes) and assessing whether, and at what range of ANI values, genomovar-discriminating valleys appear. The threshold mentioned above works well in 75% or more of the species examined thus far. Identifying and tracking these units over time or perturbations/treatments with long-read metagenomes is also achievable, e.g., long-read data have enough information to show the ANI gap as previously demonstrated.^40^ However, it should be mentioned that the ANI gap discussed above might not be observed in metagenome assembled genomes constructed in short-read metagenomic studies due to the assembly step merging highly similar sequences into a consensus (e.g., sequences sharing > 97–98% nucleotide identity). Popular tools for strain-level identification in metagenomic datasets such as inStrain and StrainGE to name a few,^49,50^ have not been adjusted to incorporate the ANI-based units reviewed above but their output can often provide a view of diversity patterns, which can be subsequently examined for gaps in diversity. Therefore, it is now possible to track the relative abundance, functionality (e.g., metatranscriptomics) and evolution (e.g., mutational events or horizontal gene transfer) of genomovars in microbiome studies and obtain results that do not have the limitations of conventional methods for intra-species resolution such as 16S rRNA gene amplicon sequencing, STs, and short-read metagenomics. Regarding the estimation of relative abundance based on mapping short- or long-read metagenomic data against reference genome(s), which typically represents a first and key step in any microbiome study, a robust approach should normalize for spurious read matches using concepts like the TAD80 (Truncated Average sequencing Depth over the middle 80% of indices sorted by depth)^51^ and metagenomic sequencing effort, but also for any substantial differences (e.g., >2 fold) in average genome size between metagenomes using MicrobeCensus.^52^ The derived relative abundance values from such an approach should be directly comparable between samples, as reviewed recently.^53^ Pangenome amplification of low-abundance populations with hybrid selection has also been shown to be useful to identify transcriptional differences among enteric *Escherichia* populations and could have broader utility for dissecting strain-level composition and function.^54^ Spiking in reference standards to convert (the default) metagenomic relative abundance into absolute abundance may also be desirable in some cases.^55^ For further discussion of the underlying causes of false-positive signals, bioinformatics tools to use for abundance estimation, types of normalizations needed under different sequencing scenarios, and how to apply them can be found in recent published work.^53^ These recent advances in terms of intraspecies resolution as well as estimation of relative abundance will significantly facilitate ongoing and future microbiome studies and interventions.

## Competition mechanisms by which gut microbiota mediate colonization resistance and their implications for MDRO decolonization

**Figure 1.**
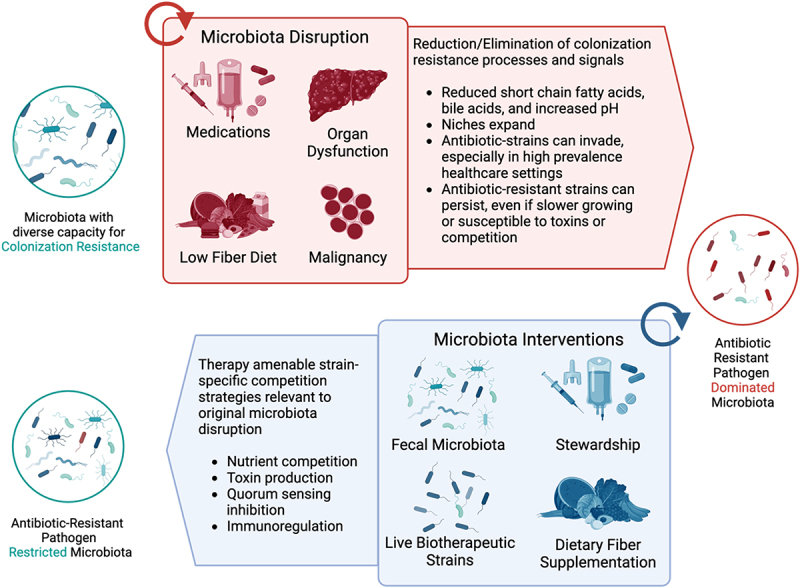
Microbial competition mechanisms that contribute to colonization resistance can be disrupted by many routine exposures and potentially restored by strain-aware microbiota interventions. In some patients, particularly with high healthcare burden, these disrupting exposures recur or progress (noted by circular arrow), which likely contributes to further impairments in restricting colonization with antibiotic-resistant strains. Basic science and translational studies highlighted in this review suggest interventions that may be used to exploit fitness costs of antibiotic resistant strains and enhance colonization resistance. From this perspective, supporting optimal prescribing of antibiotics and other disruptive medications and dietary fiber supplementation can be viewed as microbiota interventions. The ideal microbiota intervention(s) would be feasible to administer as often as indicated (also noted by circular arrow).

Leveraging the mechanisms by which indigenous gut flora mediate colonization resistance – that is, the ecological processes and dynamics that restrict colonization and dominance by pathogens^56–58^ – has potential for reducing MDRO colonization and AMR. These mechanisms include competition for nutrients, metabolites and space, direct bacterial antagonism via contact-dependent and independent mechanisms, and modulation of host immunity (Figure 1).^5,59,60^

Bacterial competition mechanisms are important for microbiota therapeutic development from the perspective of reducing fitness of an MDRO. For example, antibiotic exposures can select for isolates with high minimal inhibitory concentrations (MIC) but slower growth rates than isolates with relatively lower MICs.^61^ This slower growth rate is described as a fitness cost of antibiotic resistance. However, in some contexts, after several generations selected by continued antibiotic pressure, mutants can be isolated that are antibiotic resistant without fitness costs.^62,63^ Shifting microbiota composition may also shift fitness constraints and contribute to replacement of MDRO strains with more susceptible strains as observed in the PREMIX trial.^21^ Clearer understanding of the fitness costs of antibiotic resistance mechanisms could inform rational design of microbiota treatments to select for antibiotic susceptibility.

The prevalence of competition in the gut bacterial ecosystem presents multiple promising avenues for therapeutic strategies to reduce MDRO infection risk by undermining their colonization strategies. In the following sections, we will discuss how the mechanisms of nutrient and metabolite exclusion, quorum sensing inhibition, and direct bacterial antagonism may have therapeutic value for MDRO decolonization and the efforts that have been made to identify and characterize how bacterial species and defined bacterial communities confer colonization resistance against human pathogens.

## Differential nutrient/carbon source utilization

To establish colonization and infection, bacterial pathogens must be able to compete with the indigenous microbiota for nutrients. This resource competition has long been studied as a mechanism of colonization resistance.^64,65^ For example, in a murine model, commensal and pathogenic *E. coli* strains were demonstrated to utilize different sugars for growth.^66^ Furthermore, the preferential consumption of sugars among different commensal *E. coli* species^67^ impacted their relative abilities to exclude the colonization of other commensal *E. coli* as well the O157:H7 pathogen, *E. coli* EDL933.^68^ It would stand to reason then that pathogen exclusion should be enhanced by a commensal group with a diverse complement of nutrient needs that restrict almost all available nutritional niches. This concept was demonstrated when the mice that were pre-colonized with three commensal *E. coli* strains of varying sugar utilization profiles had lower growth of the pathogenic O157:H7 *E. coli* strain than any of the mice that were pre-colonized with the commensal strains individually.^68^

To apply this on a larger scale, microbiome analytic methods that increase the throughput of screening for these competitive interactions in complex microbial communities are much needed. Schluter and colleagues^69^ recently developed the TaxUMAP atlas and in their application, showed that certain *Klebsiella spp* associated with low bacteremia risk were localized to a region that was depleted in other enterobacteria associated with high bacteremia risk. This suggested a competitive exclusion that was validated experimentally using a phenotypic microarray analysis, which showed that the majority of carbon sources favored these *Klebsiella spp* over the high-risk enterobacteria under aerobic conditions.^69^

Using another approach, Spragge and colleagues^70^ studied the nutrient utilization effects of 100 human gut symbionts, individually and in combinations, in colonization resistance for two pathogens, *K. pneumoniae* and *Salmonella enterica* serovar Typhimurium. Using *in vitro* and *in vivo* experiments in gnotobiotic mice, they found that although colonization resistance was a complex community-level effect, it was dependent on the presence of specific keystone species in the context of overlapping nutritional requirements between the symbiont community and the pathogen.^70^ Collectively, these findings advance a framework for the rational design of strains or microbial consortia to restrict pathogen growth through nutrient consumption.

Among several important carbon sources that are associated with bacterial colonization and infection such as sialic acid, acetylglucosamine and mucins, fucose, and glycans, ethanolamine (EA), is a specific example of how metabolic activity can restrict pathogen colonization. EA is a breakdown product of the phospholipid phosphatidylethanolamine and is abundant in the human gut from both colonocyte turnover and dietary sources. EA can be used by enteric bacteria as an alternative carbon and nitrogen source,^71^ and EA utilization genes are encoded on the EA utilization (*eut*) locus. In a study that combined text mining and comparative genomics to identify novel bacterial genotype-phenotype relationships, a preponderance of genes corresponding to EA usage clustered in food-borne pathogens suggesting these pathways might be important genomic determinants of pathogenicity.^72^ EA metabolism confers a competitive advantage. This has mostly been characterized in gut bacterial pathogens^71,73–78^ but EA catabolism does not appear to be pathogen-specific as *eut* genes have been found in phylogenetically diverse bacteria, both pathogenic and nonpathogenic.^79^ In an evaluation of 40 commensal *E. coli* strains isolated from the feces of healthy donors, 35 were able to employ EA as a nitrogen and/or carbon source *in vitro*.^80^ Furthermore, when EA was provided as the sole nitrogen source, the *E. coli* commensal strains Nissle and HS were able to outcompete *E. coli* O157:H7.^81^ Contrasting with what had previously been observed for enteric pathogens, in competitive colonization experiments between a wild type *E. faecalis* and an isogenic mutant that is unable to express the *eut* genes and cannot utilize EA, the mutant had increased fitness and outcompeted its wild-type counterpart.^82^ Collectively, this indicates a more nuanced role for EA in microbiota interactions that has yet to be elucidated fully. As many other members of the gut microbiota have been shown to contain the *eut* locus,^79^ one way to further our understanding would be to characterize the extent of EA utilization by the commensal flora. In an extension of the methodology presented by de Gouveia et al.,^80^ the EA catabolism by representatives of the gut microbiome could be evaluated *in vitro* either through the measurement of growth parameters in a multi-well format when EA is used as the sole carbon source or via automated phenotypic microarray technology.^83^ EA-utilizing commensals individually and in rationally designed consortia could then be systemically evaluated for their ability to outcompete or limit colonization of pathogenic bacteria.

## Other metabolites that shape microbiota composition

Several other metabolites have been identified that can influence gut microbiota composition. Bile acids are cholesterol-derived, amphipathic molecules that are produced in the liver in their primary form, are present throughout the intestine and undergo microbial transformation to produce secondary bile acids, such as lithocholic acid and deoxycholic acid.^84,85^ Bile acids exert antimicrobial effects at high concentrations, and gut microbiota-derived secondary bile acids in particular play an important role in infection resistance.^84^ Fecal microbiota transplantation for *C. difficile* infection has been shown to change the recipient’s intestinal bile acid metabolome to reflect the metabolome of the donor, suggesting that normalization of bile acid metabolism may be a critical element for intestinal exclusion of this pathogen.^86^ Additionally, *Clostridium scindens* inhibits the growth of *C. difficile* by producing lithocholic acid and deoxycholic acid; this effect was recapitulated *in vivo* where *C. scindens* or a bacterial consortium capable of producing lithocholic and deoxycholic acid protected mice against *C. difficile* infection.^87^

However, the effect of bile acids is not unidirectional against potential pathogens. They can also facilitate pathogen intestinal colonization. An important human pathogen, vancomycin-resistant *Enterococcus* (VRE) undergoes a morphotype switch from diplococcal to chained growth that is triggered by lithocholic acid.^88^ This inducible morphotype switch promotes biofilm formation that is key for host intestinal colonization and persistence. This switch could be reversed by treatment with divalent metal cations.^88^ The ability to modulate this morphotype switch to favor the diplococcal form to facilitate clearance represents a potential target for VRE decolonization therapies.

In the lower intestinal tract, gut microbiota can produce short chain fatty acids (SCFAs) such as acetate, propionate and butyrate through fermentation of non-digestible carbohydrates.^89^ While SCFAs have an established role in maintenance of the colonic epithelium, as a colonic enterocyte energy source, and in acidification of the gut lumen,^90,91^ they can also help regulate pathogen invasion. *Bacteroides*-derived propionate inhibits *S. enterica* serovar Typhimurium expansion in the distal colon and fecal shedding^92^ and depletion of *Clostridia spp*, the primary producers of butyrate in the gut, promotes *S. enterica* serovar Typhimurium expansion.^93^ In another example, butyrate-producing bacteria were found to be necessary for protecting mice against *C. rodentium* infection, and butyrate supplementation of susceptible, germ-free mice decreased the burden of *C. rodentium* colonization.^94^

Physiological concentrations of SCFAs also inhibit the expansion of antibiotic-resistant *E. coli*, *K pneumoniae*, and *P. mirabilis* isolates in a pH-dependent manner through a mechanism of SCFA-mediated intracellular acidification.^95^ In the same investigation, the authors showed that in a patient with a history of allogeneic hematopoietic stem cell transplantation, increasing antibiotic exposure was associated with a loss of SCFAs and a disproportionately dense representation of *E. coli* in the patient’s gut microbiota that preceded bloodstream infection with this organism. Taken together, this suggests that SCFA modulation may be a potential way to alter susceptibility to pathogen colonization and subsequent infection.

## Cross-species inhibition of quorum sensing

Another strategy to exploit mechanisms of colonization resistance is interruption of pathogen signaling systems that facilitate growth and virulence such as quorum sensing. Quorum sensing (QS) describes chemical communication between cells via the secretion of diffusible small molecules, known as autoinducers, by individual cells that leads to coordinated population behavior. Both intraspecies and interspecies quorum sensing have been described.^96–98^ *E. coli* strains engineered to alter the levels of the interspecies QS autoinducer-2 (AI-2) molecule in a mouse model of antibiotic-induced gut dysbiosis showed that increased levels of AI-2 helped reestablish a healthy gut microbiome by favoring the expansion of Firmicutes over Bacteroidetes.^99^ An interspecies interaction between the oral probiotic *Streptococcus salivarius* K12 and *Streptococcus pyogenes*, also known as group A *Streptococcus* (GAS), is mediated by a quorum sensing peptide. *S. salivarius* uses its quorum sensing peptide to control the production of an antibiotic known as salivabactin that has potent anti-GAS activity. In turn, *S. pyogenes* subverts the probiotic quorum sensing signal to activate the production of a secreted protease that leads to degradation of the probiotic antimicrobial peptides such that GAS survival occurs during dual species growth. A re-engineered *S. salivarius* K12 that had an inactivation of the peptide that led to secreted protease production in GAS as well as a constitutively active promoter that led to augmented salivabactin production inhibited GAS colonization *in vivo*. ^100^

Interruption of quorum sensing to limit intestinal colonization as a source for infection has also been demonstrated for the important human pathogen *S. aureus*. While nasal carriage of *S. aureus* is a well-known risk factor for infection,^101,102^ *S. aureus* has also been isolated from other body sites, and intestinal colonization likely represents an important reservoir for this pathogen that has been underappreciated.^103–106^ A culture-based analysis found a strong negative correlation between the presence of *Bacillus* spp in the gut and intestinal and nasal colonization by *S. aureus*. ^107^ In follow-up analyses, it was shown that fengycins, a class of *Bacillus* lipopeptides, inhibit the *S. aureus* Agr QS signaling system and that feeding fengycin-producing bacterial spores to mice led to eradication of *S. aureus* intestinal colonization. This work was extended to determine the robustness of this decolonization effect in humans. In a single-center phase 2 clinical trial conducted in Thailand, healthy participants with documented *S. aureus* colonization were randomized to receive *B subtilis* probiotic versus placebo with the primary outcome of nasal and gut *S. aureus* colonization after 30 days of intervention.^108^ In the probiotic group, there was a 96.8% reduction of *S. aureus* carriage density in the stool and a 65.4% reduction in the nose without significant alterations in the gut microbiome between the placebo and intervention groups. Mechanistically, these studies also demonstrate that inhibition of quorum sensing systems is a viable method for reducing the burden of pathogen colonization.

Several knowledge gaps prevent leveraging quorum-sensing inhibition as a microbiome therapeutic target more broadly. Annotation of genes involved in quorum sensing, much less experimental validation in the context of microbiomes, has not yet matured to the point to allow high-throughput screens that are typical of metagenomic studies. The Quorum Sensing of Human Gut Microbes (QHSGM) database was developed by Wu and colleagues to help address this deficiency.^109^ The continued development of structured classification criteria of quorum-sensing genes in databases and the quorum-sensing microbe-microbe networks predicted by said databases would accelerate study of the role of quorum sensing in MDRO colonization and its broader potential for interruption with microbiome therapies.

## Bacteriocins

Given the prevalence and diverse repertoire of attack mechanisms that have been characterized in gut symbionts, direct interbacterial antagonism is thought to be pervasive and important for shaping gut microbiota structure and composition.^60,110,111^ One such mechanism of direct inhibition is the elaboration of diffusible toxins, such as bacteriocins. Bacteriocins are a heterogeneous group of diffusible, ribosomally synthesized antimicrobial peptides that exert their killing activity through a variety of different mechanisms, including pore formation, peptidoglycan synthesis inhibition, and interference with DNA, RNA and protein metabolism.^112^ They are produced across all major groups of bacteria. The antimicrobial spectrum of activity is typically narrow, targeting species or genera that are closely related to the producing strain though lactococcal nisin is unusual for its broad inhibitory spectrum against a variety of gram-positive bacteria.^113^ Historically, the majority of bacteriocins were identified through labor-intensive culture-dependent screening methods and biochemical characterization.^114,115^ With advances in sequencing technology and the growth of sequence databases, several tools now exist to detect biosynthetic gene clusters in assembled bacterial genomes.^116–118^

There are numerous examples of the *in vivo* protective effect of bacteriocin-producers against pathogens. *L. salivarius* UCC118 inhibits infection *with L. monocytogenes* in mice through the production of a two-peptide bacteriocin.^119^ Similarly, oral administration of human stool-derived bacteriocin-producing strains of *Lactococcus lactis* MM19 and *Pediococcus acidilactici* MM33 decreased the intestinal VRE population in mice^120^ and intraperitoneal administration of the *S. mutans*-derived-lantibiotic mutacin B-Ny266 was effective at preventing mortality in a mouse model of intraperitoneal methicillin-susceptible *S. aureus* infection.^121^ An investigation of the probiotic potential of bacteriocin-producing commensal *E. coli* strains against pathogenic porcine ETEC and STEC showed that treatment of piglets with a cocktail of three bacteriocinogenic *E. coli* isolates of human origin resulted in decreased duration of pathogen shedding and diarrhea.^122^ In a dextran sulfate sodium-treated mouse model of colitis, the ability of *E. coli* Nissle 1917 to produce microcins, a subclass of bacteriocins with narrow spectrum, limited expansion of its competitors, including related commensal *E. coli*, AIEC, and the pathogen *S. enterica* serovar Typhimurium.^123^ *E. faecalis* isolates harboring the conjugative plasmid pPD1 that encodes the enterococcal bacteriocin Bac-21 outcompete E. *faecalis* that do not have the plasmid in competitive colonization experiments in mice.^124^ Bac-21 was shown to have *in vitro* activity against a panel of multidrug-resistant clinical isolates of *E. faecalis* and *E. faecium* and, notably, in mice colonized with vancomycin-resistant *E. faecalis* (VRE) strain, challenge with a Bac-21+ *E. faecalis* strain with a conjugation defect (to eliminate the risk of bacteriocin transfer) also resulted in decolonization of the VRE.

Bacteriocins are an appealing therapeutic because their peptidic nature makes them amenable to modification through gene-based peptide engineering and/or chemical synthesis techniques to produce derivatives with enhanced features such as modulated spectrum of activity, increased potency and increased stability.^125–132^ There is potential to fine-tune bacteriocins with a desired antimicrobial spectrum to selectively modify the gut microbial community and/or to displace a target pathogen. Already, probiotic bacteria have been engineered to produce bacteriocins that have therapeutic activity against pathogens of interest in animal models of infection.^133–136^ Despite this success, there are limited studies on the possible off-target effects of introducing a non-native bacteriocin-producing strain on the gut ecosystem as a whole and there are concerns that targeted removal of a certain species could lead to unintended downstream effects.^137–139^ However, a study comparing the effects of five different bacteriocin producers, along with their isogenic non-bacteriocin-producing counterparts serving as reference strains, showed that overall gut microbiota structure at the phyla level was largely unperturbed by the bacteriocin producers versus non-producers though there were changes at lower taxonomic levels that corresponded to the *in vitro* inhibitory spectra of the bacteriocin.^140^ Thus, in order to realize the full therapeutic potential of bacteriocins, an understanding of their microbiome-wide effects in various contexts will be needed.

An additional concern is that bacteriocin production imparts a fitness cost, and high levels of bacteriocin production may offset the competitive advantage. In a study investigating the effect of differing transcriptional levels (wild type, low, high) of microcin C in the probiotic strain *E. coli* Nissle, there was comparable antibacterial efficacy across all three expression levels. However, the bacteria that produced the lowest amount of microcin C displayed better growth and viability characteristics and conferred the best protective effect in a *Galleria mellonella* model of enterohemorrhagic *E. coli* infection.^141^ These findings demonstrate that high amounts of bacteriocin are not necessary to achieve the desired antibacterial effect, which could inform therapeutic design and dosing.

## Type VI secretion systems

Among the contact-dependent inhibition systems, there is increasing evidence that the type VI secretion system (T6SS) plays a particularly important role in gut microbiome community composition and structure.^142,143^ The T6SS is a contact-dependent form of antagonism in which an attacking cell uses a contractile apparatus to inject effector proteins through the membrane of a target organism.^144^ The effector protein is usually produced with a cognate immunity protein which protects the attacking cell and its sister cells from the toxicity of its own effector.^145,146^ The T6SS is widely distributed in gram-negative bacteria, estimated to be present in approximately 25% of gram-negatives.^143,147,148^ The deployment of T6SS to overcome colonization resistance and establish infection has been documented in the enteric bacterial pathogens *S. enterica* serovar Typhimurium, *Vibrio cholerae*, and *Shigella sonnei*.^149–151^ However, the effect of T6SS is not unidirectional, and resident microbiota also employ T6SS to counter pathogen invasion and reinforce colonization resistance. In a manner akin to dueling, the murine pathogen *C. rodentium* uses one of its T6SS to target and outcompete a commensal *E. coli strain* to establish residency in the murine gut, which is thwarted by the deployment of that commensal’s own T6SS.^152^

Further, there is broad conservation of T6SS loci in the Bacteroidetes,^153,154^ one of two dominant phyla that comprise the human gut microbiome.^155^ Mathematical, bioinformatic and functional characterization suggests that T6SS’s can shape gut microbial community composition and can be important determinants of colonization resistance by enabling the temporal stability and persistence of human gut symbionts.^142,153^ In a mouse model, strain competition between introduced co-cultures of non-toxigenic and enterotoxigenic strains of *B. fragilis* was found to be mediated by T6SS, and a functional T6SS was a requirement for full protection of the murine host from toxin-induced colonic injury.^156^ A potential therapeutic strategy against enterotoxigenic *B. fragilis*-mediated disease might be re-colonization with genetically modified non-toxigenic strains through microbiota-based therapeutics. More importantly, this finding highlights the importance of further examining the generality of T6SS-mediated competitive exclusion in other pathogens that have both toxigenic and non-toxigenic counterparts, such as *E. coli* or *C. difficile*. While more work needs to be done demonstrating the role of T6SS in reducing pathogen colonization for clinical bacterial isolates and human subjects, supplementation of T6SS-carrying bacteria that target specific pathogens seems a promising strategy for MDRO decolonization. Indeed, there have already been attempts to exploit T6SS to selectively deplete target bacteria. Using nano-body based cell-cell adhesion technology to promote the specific adhesion of a T6SS-carrying bacterium to a target cell, Ting et al were able demonstrate selective eradication of a target *E. coli* from complex polymicrobial assemblages without significant off-target effects.^157^

## The impact of gut microbiota composition on colonization resistance

Despite greater understanding of the mechanisms above, the necessary components for microbiota protection are just beginning to be uncovered. Harnessing the protective power of the gut microbiome requires a detailed understanding of the taxa and the functions that confer protection against specific pathogens. Identification and manipulation of minimal commensal consortia that collectively exclude a pathogen of interest represents a practical therapeutic avenue for MDR decolonization. Several members of the enteric microbiota have defined protective roles against important pathogens^158^ and this section will review recent advances in competitive exclusion at the community/microbial consortium level.

In genetically similar mice from different commercial vendors, heterogeneity in susceptibility to *S. enterica* serovar Typhimurium infection was related to underlying gut microbiota variation.^159^ Similarly, isogenic mouse lines with diverse microbiota compositions also significantly differ in their susceptibility to *C. rodentium* infection. Microbiome and metabolomic profiling linked the resistant phenotype to elevated SCFA, particularly butyrate, production by indigenous microbiota. Butyrate supplementation *in vitro* and *in vivo* recapitulated the resistant phenotype.^94^ Using a modular design approach to define the minimal bacterial consortium necessary for colonization resistance against *S. enterica* serovar Typhimurium, Brugiroux and colleagues identified the Oligo-Mouse-Microbiota (Oligo-MM^12^) a synthetic community of 12 sequenced and publicly available strains isolated from mice, representing the five bacterial phyla that are naturally abundant in the mouse GI tract.^160^ Oligo-MM^12^ members lack the enzymatic pathway responsible for 7α-dehydroxylation, which is critical for the generation of secondary bile acids, and this consortium is unable to impart colonization resistance against *C. difficile*. The addition of *C. scindens*, which possesses this critical pathway, restored 7α-dehydroxylation functional capability to the consortium as a whole and led to partial protection from *C. difficile* infection.^161^

Bacteroidetes have been shown to protect against *K. pneumoniae* intestinal colonization via indirect immune stimulation. In this process, Bacteroidetes associate with the mucosal barrier and prime intestinal IL-36γ secretion, which promotes bactericidal activity of macrophages against multiple strains of *K. pneumoniae*.^162^ In other work by Nagao-Kitamoto et al, host IL-22 production is induced by the gut microbiota, which regulates host glycosylation^163^ and supports growth of *Phascolarctobacterium spp*, which consume succinate and prevent *C. difficile* growth. By comparing mouse microbiota that provide different levels of protection against VRE colonization, a minimal four-member commensal consortium comprised of *Clostridium bolteae*, *Blautia producta*, *Bacteroides sartorii*, and *Parabacteroides distasonis* was identified that is protective against VRE.^164^ Within this consortium, *B. producta* is directly antagonistic to VRE through the elaboration of a lantibiotic that disproportionately inhibits VRE compared to gram-positive commensal species.^165^

Honda et al^166^ characterized the *Klebsiella*-decolonizing capacity of commensals from the feces of five healthy donors. Using a down-selection strategy, they were able to identify a minimal consortia of 18 strains from one of these donors whose colonization resistance against MDR Klebsiella was mediated through restriction of gluconate availability.^166^ In a *post-hoc* analysis of adults with recurrent *C. difficile* infection and colonized with *Pseudomonadota*, the administration of a 33-member microbial consortium called MET-2 was associated with *Pseudomonadota* decolonization and an increase in the abundance of obligate anaerobes and butyrate-producers.^167^ Using a combination of metagenomics, metatranscriptomics, targeted metabolomics and mouse models, a 5-member consortium consisting of *Alistipes*, *Barnesiella*, *Olsenella*, *Oscillibacter* and *Flavonifractor* reduced VRE colonization in antibiotic-treated mice. This effect was due to the consortium’s ability to deplete fructose, which was later found to be driven primarily by the *Olsenella*.^168^

Multiple reports have described the importance of the *Enterobacteriaceae*, a typically low abundance taxon in the gut microbiome, in colonization resistance that occurs via aerobic respiration-dependent differential carbon source utilization and resource competition.^70,159,169^ In a study investigating the competition of commensal *Enterobacteriaceae* with *Salmonella enterica* serovar Enteriditis in the guts of neonatal chicks, full colonization resistance against this pathogen required the presence of both anaerobic spore-forming bacteria and *Enterobacteriaeceae* and could not be achieved by using *Enterobacteriaeceae* alone.^169^ The colonization resistance of *E. coli* against *S. enterica* serovar Enteriditis is thought to be mediated by the former’s consumption of oxygen, which is a critical resource for the latter. This effect is exacerbated in the presence of anaerobic spore-forming bacteria, who in the course of maintaining luminal epithelial hypoxia, make oxygen a limiting resource.^169^ On this note, the importance of anaerobic bacteria cannot be overstated. In the course of designing Oligo-MM^12^, colonization resistance against *S. enterica* serovar Typhimurium required the inclusion of facultative anaerobic bacteria.^160^ Similarly, obligate anaerobic bacteria are important for VRE clearance. Anaerobically cultured fecal pellets were just as effective as unfractionated stool at reducing VRE colonization in mice whereas aerobically cultured fecal pellets did not result in VRE elimination. The former effect was later ascribed to the presence of the obligate anaerobic genus *Barnesiella*. ^170^ The continued expansion of our understanding of the individual contributions of certain taxa to colonization resistance is critical to identifying which ones should be prioritized for inclusion when designing future generations of microbiome-based therapeutics.

## Conclusion

Mounting work is uncovering specific processes by which intestinal microbiota can restrict pathogen colonization. In turn, a restricted pathogen niche increases competitive pressure between low-abundance strains. Evidence of this competitive pressure is identifiable as selection for strain-specific adaptations, including resistance or immunity to toxins like T6SS and bacteriocins, metabolism of available carbon sources, tolerance of secondary bile acids and short chain fatty acids, and in some cases, selection against AMR genes, particularly when they carry a fitness cost. Although strain competition mechanisms and microbiota interventions outlined here have been observed in a limited number of human studies, they show clear potential to reduce the overall carrying capacity for MDROs, select against MDRO strain dominance, or even potentially revert to antibiotic susceptible phenotypes.^21,69^ The mechanistic insights from these studies should motivate further translational studies to determine whether there are certain biomarkers, such as baseline microbial community composition or metabolite production, that are predictive of those who would be at most risk for MDRO colonization or most likely to benefit from microbiome therapy.

A limitation of these studies is the paucity of data regarding the microbiome-wide impact of these interventions given the strong interdependency of bacterial ecological networks. Clearly, much work remains to be done to evaluate the impact of strain-specific therapeutic strategies alone or in combination. At steady state, many microbial communities are resilient to many perturbations. However, even biomolecules with an apparent narrow spectrum of activity could disrupt microbiota composition more broadly by reducing taxa that support other beneficial taxa through cross-feeding, spatial proximity, and other interactions. This potential safety issue may not be identifiable without larger clinical studies of diverse patients of varied baseline gut microbiota composition.

Understanding how the intestinal microbiota directly influences MDRO colonization also clarifies how dramatically disruptive routine healthcare can be to these systems and how this disruption can compound, particularly among patients who require frequent healthcare encounters. Two thirds of hospitalized patients^171–173^ are administered broad spectrum antibiotics, and many patients experience large shifts in diet that has independently been linked to SCFA production and expansion of pathogens.^174–176^ These routine microbiota disruptions present clear opportunities to simultaneously answer important questions in microecology and expand treatment options for patients. Microbiota disruption related to healthcare is also amenable to study of pragmatic interventions such as antibiotic stewardship programs, fiber supplementation, and natural history clinical studies that better reflect the experience of patients with frequent healthcare exposures than might be feasible to assess in mouse models or healthy participant studies.

Looking forward, several gaps remain in leveraging this growing awareness of AMR strain dynamics within the gut to develop microbiota therapies for AMR. Three areas may be suggested as priorities. First, biotherapeutic strains would benefit from rigorous engraftment studies, ideally controlled by placebo and FMT treatment groups, to determine optimal regimens for recipient conditioning (to clarify risks and benefits of pre-treatment with antibiotics or bowel preparation), dose, and route of administration. These data are crucial to inform more efficient trial design, avoid incorrect or premature efficacy determinations if a trial endpoint is not met due to underdosing, and to optimize potential benefit to trial participants.^25^ Second, large observational cohort studies are needed to nominate and validate microbiota-informed biomarkers for colonization resistance, MDRO colonization, shedding, and progression from colonization to infection. These data could advance development of much needed surrogate endpoints to support clinical trials and potentially clinical practice. An example of how this could be applied is through design of clinical trials that screen and enroll by given stool metabolite concentrations, in which participants are treated with biotherapeutics that can boost these metabolites, with or without diet or prebiotic supplementation. Third, it remains unclear whether colonization resistance that is robust to invasion by AMR strains is an emergent property or can be established with a minimal subset of therapeutic strains.^177^ A longer-term goal is to address this question in studies stratified by disrupting exposure (e.g. antibiotic class) and priority AMR class or classes. Addressing these priority areas has the potential to enhance microbiota therapeutic efficacy, reduce incidence of infection, and improve outcomes for patients and populations.

## Data Availability

Data sharing is not applicable to this article as no new data were created or analyzed in this study.
